# Scar Prevention and Enhanced Wound Healing Induced by Polydeoxyribonucleotide in a Rat Incisional Wound-Healing Model

**DOI:** 10.3390/ijms18081698

**Published:** 2017-08-03

**Authors:** Woonhyeok Jeong, Chae Eun Yang, Tai Suk Roh, Jun Hyung Kim, Ju Hee Lee, Won Jai Lee

**Affiliations:** 1 Department of Plastic and Reconstructive Surgery, School of Medicine & Institute for Medical Science, Keimyung University, Dongsan Medical Center, Daegu 41931, Korea; psjeong0918@gmail.com (W.J.); med69@dsmc.or.kr (J.H.K.); 2 Department of Plastic and Reconstructive Surgery, Institute for Human Tissue Restoration, Yonsei University Health System, Severance Hospital, Seoul 03722, Korea; cheniya@yuhs.ac; 3 Department of Plastic and Reconstructive Surgery, Institute for Human Tissue Restoration, Gangnam Yonsei University Health System, Severance Hospital, Seoul 06273, Korea; tsroh@yuhs.ac; 4 Department of Dermatology and Cutaneous Biology Research Institute, Severance Hospital, Yonsei University College of Medicine, Seoul 03722, Korea

**Keywords:** polydeoxyribonucleotide, cicatrix, inflammation, wounds and injuries, rats

## Abstract

High-mobility group box protein-1 (HMGB-1) plays a central role in the inflammatory network, and uncontrolled chronic inflammation can lead to excessive scarring. The aim of this study was to evaluate the anti-inflammatory effects of polydeoxyribonucleotide (PDRN) on scar formation. Sprague-Dawley rats (*n* = 30) underwent dorsal excision of the skin, followed by skin repair. PDRN (8 mg/kg) was administered via intraperitoneal injection for three (PDRN-3 group, *n* = 8) or seven (PDRN-7 group, *n* = 8) days, and HMGB-1 was administered via intradermal injection in addition to PDRN treatment for three days (PDRN-3+HMGB-1 group; *n* = 6). The scar-reducing effects of PDRN were evaluated in the internal scar area and by inflammatory cell counts using histology and immunohistochemistry. Western blot, immunohistochemistry and immunofluorescence assays were performed to observe changes in type I and type III collagen and the expression of HMGB-1 and CD45. Treatment with PDRN significantly reduced the scar area, inflammatory cell infiltration and the number of CD45-positive cells. In addition, the increased expression of HMGB-1 observed in the sham group was significantly reduced after treatment with PDRN. Rats administered HMGB-1 in addition to PDRN exhibited scar areas with inflammatory cell infiltration similar to the sham group, and the collagen synthesis effects of PDRN were reversed. In summary, PDRN exerts anti-inflammatory and collagen synthesis effects via HMGB-1 suppression, preventing scar formation. Thus, we believe that the anti-inflammatory and collagen synthesis effects of PDRN resulted in faster wound healing and decreased scar formation.

## 1. Introduction

Inflammation is an inevitable first step in the process of wound healing and is closely related to scar formation. However, continuous and chronic inflammation stimulates the secretion of pro-inflammatory cytokines and causes excessive scarring [1,2,3,4]. In one prior study involving a scarless fetal wound-healing model, scar formation was caused by the injection of mast cells [5]. In addition, despite the observation that neutrophil depletion did not alter wound-breaking strength or collagen deposition, neutrophil depletion resulted in wounds that healed in a more organized fashion compared with normal wounds [6]. Furthermore, another previous study revealed that macrophage depletion also reduced scar formation [7]. Hence, we hypothesized that the inhibition of inflammatory cell infiltration could be a factor in reducing scar formation.

The sustained infiltration of immune cells during prolonged and intense inflammation contributes to the continuous growth of keloid lesions [8,9]. Moreover, keloid growth involves an abnormal response to inflammation [4,10,11]. In particular, extracellular high-mobility group box protein-1 (HMGB-1) plays a central role in the inflammatory network, as it is induced by a number of cytokines and can in turn induce a series of inflammatory reactions [12]. HMGB-1 can stimulate inflammation by binding to several receptors and acts as a potent inflammatory cytokine [13]. Although there have been few studies on the relationship between HMGB-1 and fibrosis or scarring, the serum level of HMGB-1 has been positively correlated with skin thickness in systemic sclerosis [14]. Furthermore, icariin, which is used to treat erectile dysfunction, has been shown to reduce liver fibrosis in a thioacetamide-induced liver fibrosis model by antagonizing the increase in HMGB-1 in addition to other mechanisms [15].

Polydeoxyribonucleotide (PDRN) is composed of a mixture of nucleotides extracted from trout sperm. PDRN exerts anti-inflammatory effects by inhibiting mast cell degranulation and inflammatory cytokines [16,17]. A previous study reported that PDRN administration reduced pro-inflammatory mediators, such as tumor necrosis factor alpha (TNF-α), interleukin 6 (IL-6), and HMGB-1 [18]. Although it could be hypothesized that PDRN may reduce scarring by down-regulating inflammatory reactions and HMGB-1, no previous studies have investigated the relationship between PDRN and scarring. Accordingly, the aim of this study was to evaluate the anti-inflammatory effects of PDRN, including reduced infiltration of inflammatory cells and HMGB-1 expression, on scar formation via short-duration administration.

## 2. Results

### 2.1. Polydeoxyribonucleotide Decreases Scar Size in Incisional Scar Tissue in Rats

**Figure 1:**
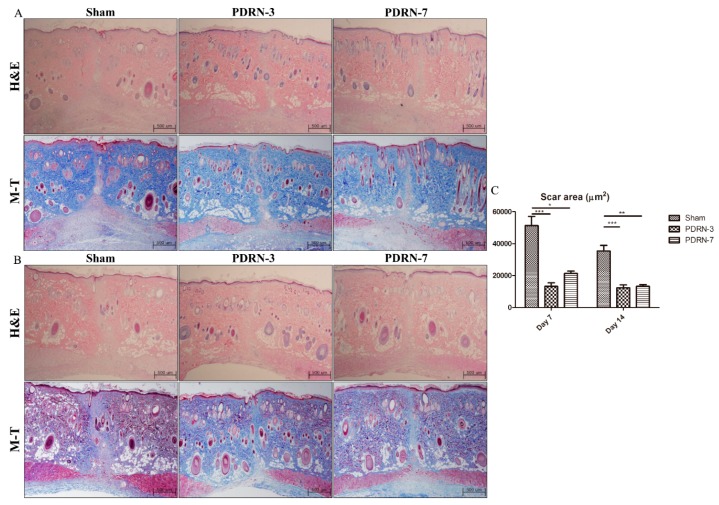
H&E- and M-T-stained tissues from the sham, Polydeoxyribonucleotide (PDRN)-3, and PDRN-7 groups on Days 7 and 14 (magnification, 40×). (**A**) All groups exhibited complete re-epithelialization and the formation of granulation tissue on Day 7. However, the PDRN-3 group showed the narrowest granulation tissue area among all groups. (**B**) Hematoxylin and eosin (H&E)- and Masson’s trichrome (M-T)-stained tissues from the sham, PDRN-3, and PDRN-7 groups on Day 14 (magnification, 40×). The sham group continued to show a wide granulation tissue area with inflammation. However, the PDRN-3 and PDRN-7 groups showed more collagen deposition within narrower scar areas, as demonstrated by M-T staining. (**C**) Quantitative analyses of the scar areas in each treatment group. The scar areas were significantly narrower in the PDRN-3 and PDRN-7 groups than in the sham group on Days 7 and 14. However, no significant difference in scar size was observed between the PDRN-3 and PDRN-7 groups on Day 7 or 14 (* *p* < 0.05, ** *p* < 0.01, *** *p* < 0.001).

**Figure 2:**
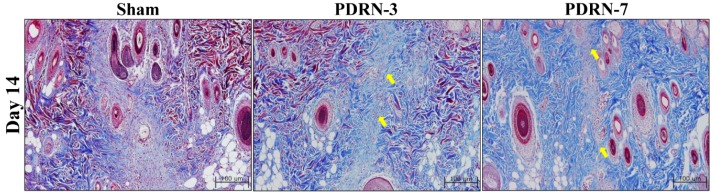
The sham group still exhibited granulation tissue and fewer collagen fibers with inflammatory cell infiltration on Day 14. However, the PDRN-treated groups demonstrated reduced inflammatory cell infiltration and collagen fibers (arrows) within the scar area.

On Day 7 of the postoperative period, all groups exhibited complete re-epithelialization and the formation of granulation tissue, as demonstrated by hematoxylin and eosin (H&E) and Masson’s trichrome (M-T) staining (Figure 1A). Although the sham group displayed active inflammation with abundant inflammatory cells and fewer collagen fibers on Day 14 of the postoperative period, the PDRN-3 and PDRN-7 groups exhibited lower inflammatory cell infiltration with collagen fibers in the scar area in the H&E- and M-T-stained tissues (Figure 1B and Figure 2).

To estimate the scar area and the degree of granulation tissue formation, only the boundary of the scar area that covered below the epidermis and above the panniculus carnosus was measured. In each wound, the scar and/or granulation tissue areas were estimated from two H&E-stained tissue sections representing different areas of the same wound. Each measurement is shown as the mean ± SEM. In the quantitative analysis of the scar area, the scar sizes of the sham, PDRN-3, and PDRN-7 groups were 51,272 ± 5793 µm^2^, 13,201 ± 2243 µm^2^, and 21,329 ± 1518 µm^2^, respectively, on Day 7 of the postoperative period (* *p* < 0.05, *** *p* < 0.001; Figure 1C), while, on Day 14 of the postoperative period, the scar sizes decreased to 35,368 ± 3511 µm^2^, 12,304 ± 1842 µm^2^, and 13,291 ± 1076 µm^2^, respectively (** *p* < 0.01, *** *p* < 0.001; Figure 1C). These results indicated that PDRN administration reduced the scar size compared with the sham group.

### 2.2. Polydeoxyribonucleotide Decreases Inflammatory Cell Infiltration in Incisional Scar Tissue in Rats

**Figure 3:**
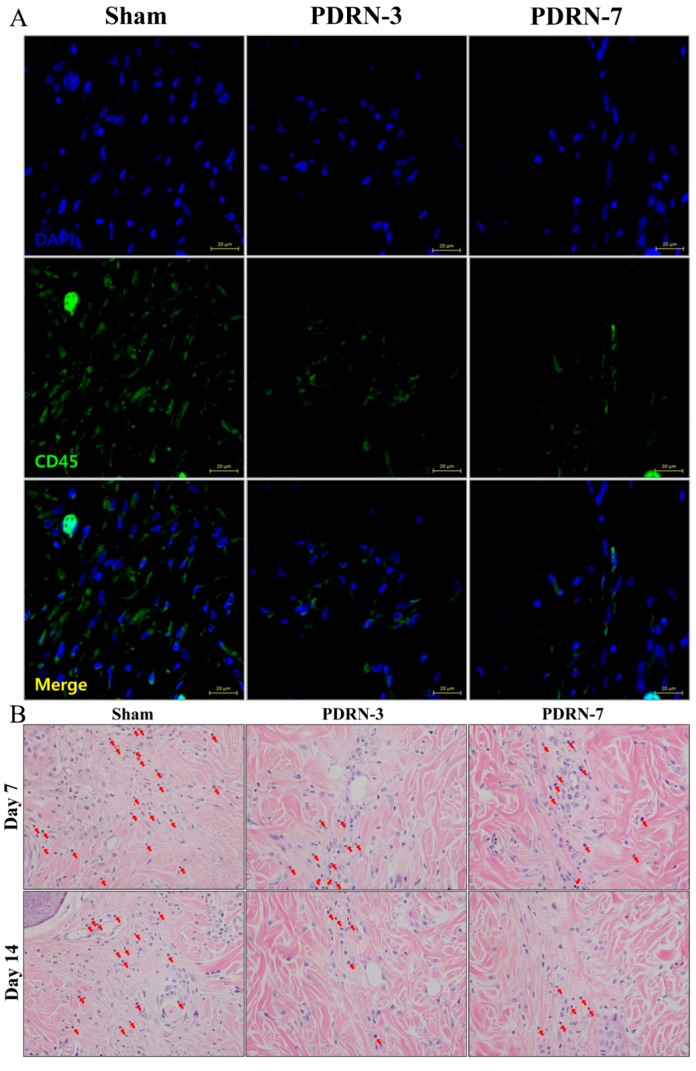

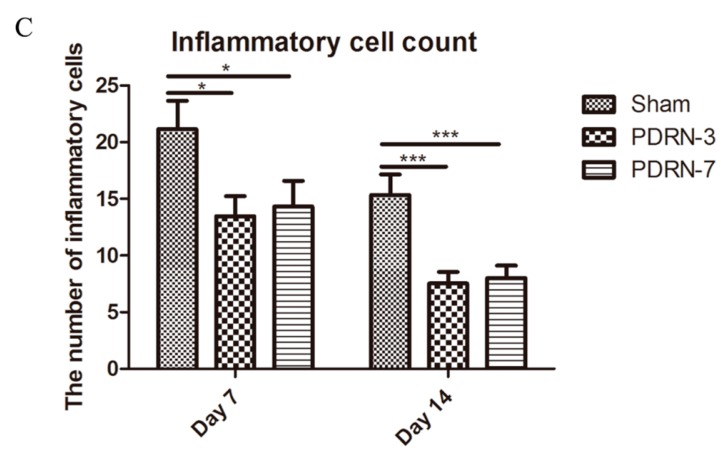
(**A**) Immunofluorescence analysis of CD45-expressing leukocytes on Day 7 (magnification, 400×). The sham group exhibited abundant cellular infiltration, and the majority of the cells were CD45-positive (green) leukocytes. The PDRN-3 and PDRN-7 groups exhibited diminished cellular infiltration and CD45-positive leukocytes. (**B**) Inflammatory cell infiltration in the scar area, as demonstrated by H&E staining (magnification, 400×). On Days 7 and 14, the sham group exhibited significant inflammatory cell infiltration into the scar area compared with the PDRN-3 and PDRN-7 groups. (**C**) Comparison of inflammatory cell counts. The infiltration of inflammatory cells in the scar area was lower in the PDRN-treated groups than in the sham group on Days 7 and 14 (* *p* < 0.05, *** *p* < 0.001).

To observe inflammatory cell infiltration within the scar tissue, we performed CD45 immunofluorescence staining on tissue collected on postoperative Day 7. More CD45-expressing leukocytes were detected in the sham group than in the PDRN-3 and PDRN-7 groups (Figure 3A). The number of inflammatory cells was calculated from four serial H&E-stained tissue sections from within the dermis of the scar area (Figure 3B). On Day 7 of the postoperative period, the mean numbers of inflammatory cells within the scar tissue were 21.16 ± 2.49, 13.47 ± 1.77, and 14.31 ± 2.28 in the sham, PDRN-3, and PDRN-7 groups, respectively. On Day 14 of the postoperative period, the mean numbers of inflammatory cells within the scar tissue were 15.34 ± 1.81, 7.53 ± 1.02, and 8.00 ± 1.12 in the sham, PDRN-3, and PDRN-7 groups, respectively. The numbers of inflammatory cells on Days 7 and 14 were significantly lower in the PDRN-3 and PDRN-7 groups than the sham group (* *p* < 0.05, *** *p* < 0.001; Figure 3C).

### 2.3. Polydeoxyribonucleotide Decreases HMGB-1 Expression in Incisional Scar Tissue in Rats

**Figure 4:**
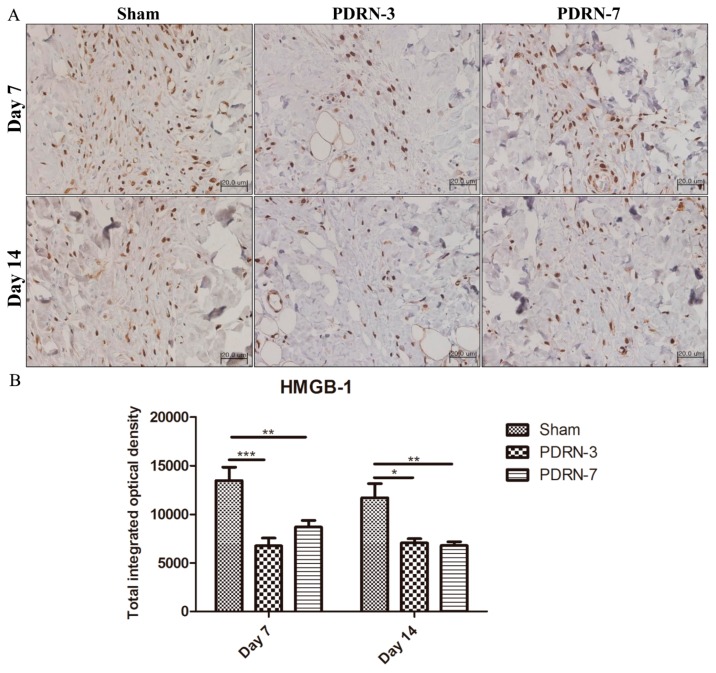
(**A**) Immunohistochemistry of high mobility group box-1 (HMGB-1) (magnification, 400×). All groups exhibited extracellular expression of HMGB-1, though the PDRN-3 and PDRN-7 groups displayed weaker expression within the narrow scar areas on Day 7. On Day 14, the sham group continued to show high extracellular expression of HMGB-1 in the wide scar areas, whereas extracellular expression of HMGB-1 was absent in the PDRN-3 and PDRN-7 groups. (**B**) Semi-quantitative analysis of HMGB-1 expression levels. The sham group showed significantly higher levels of HMGB-1 expression than the PDRN-3 and PDRN-7 groups on Days 7 and 14 (* *p* < 0.05, ** *p* < 0.01, *** *p* < 0.001).

Inflammatory cells were clearly observed within the scar tissue following staining for high mobility group box-1 (HMGB-1). On Day 7 of the postoperative period, increased HMGB-1 protein expression was observed in the sham group, whereas the PDRN-3 and PDRN-7 groups exhibited decreased HMGB-1 protein expression within the narrow scar areas. On Day 14 of the postoperative period, the sham group continued to exhibit high HMGB-1 expression in the wide scar areas, whereas the PDRN-3 and PDRN-7 groups showed markedly decreased HMGB-1 expression and only a small number of inflammatory cells (Figure 4A). Semi-quantitative analysis indicated that, on Days 7 and 14, the PDRN-3 and PDRN-7 groups exhibited significantly lower HMGB-1 protein expression than the sham group (* *p* < 0.05, ** *p* < 0.01, *** *p* < 0.001; Figure 4B).

### 2.4. HMGB-1 Administration Reverses the Anti-Inflammatory and Collagen Synthesis Effects of PDRN

**Figure 5:**
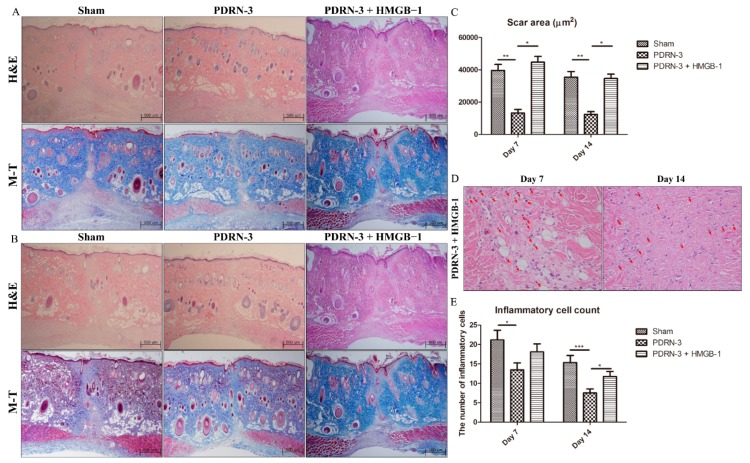
(**A**) H&E- and M-T-stained tissues from the sham, PDRN 3 and PDRN-3 + HMGB-1 groups on Day 7 (magnification, 40×). The PDRN-3 + HMGB-1 group exhibited wider scars compared with the PDRN 3 group. (**B**) H&E- and M-T-stained tissues from the sham, PDRN 3 and PDRN-3 + HMGB-1 groups on Day 14 (magnification, 40×). Although the rats in the PDRN-3 + HMGB-1 group were treated with PDRN, this group exhibited wide scars similar to the sham group on Day 14. The scar-narrowing effect of PDRN was reversed by administration of HMGB-1. (**C**) The scar areas in the PDRN-3 + HMGB-1 group were significantly wider than those in the PDRN-3 group. Additional administration of HMGB-1 reversed the scar-narrowing effect of PDRN (* *p* < 0.05, ** *p* < 0.01). (**D**) Inflammatory cell infiltration (arrow) in the PDRN-3 + HMGB-1 group, as demonstrated by H&E staining (magnification, 400×). Many inflammatory cells had infiltrated the scar area on Days 7 and 14. (**E**) The total inflammatory cell count was significantly higher in the PDRN-3 + HMGB-1 group than in the PDRN-3 group on Day 14 (* *p* < 0.05, *** *p* < 0.001). This result indicated that the anti-inflammatory effect of PDRN was reversed by HMGB-1 administration.

We next examined whether HMGB-1 administration could reverse the effects of PDRN. On Day 7 of the postoperative period, the sham and PDRN-3 + HMGB-1 groups exhibited wider granulation tissue areas than did the PDRN-3 group (Figure 5A). The sham and PDRN-3 + HMGB-1 groups continued to show higher inflammation with wider scar areas on Day 14 of the postoperative period (Figure 5B). Quantitative analysis of the scar area indicated that the scar sizes in the PDRN-3 + HMGB-1 group were 44,688 ± 3573 µm^2^ and 34,593 ± 2751 µm^2^ on Days 7 and 14, respectively. Furthermore, the scar sizes in the PDRN-3 + HMGB-1 group were similar to those of the sham group and significantly wider than those of the PDRN-3 group on Days 7 and 14 (* *p* < 0.05, ** *p* < 0.01; Figure 5C). Administration of HMGB-1 to the PDRN-3 group resulted in enhanced inflammation (Figure 5D). The mean numbers of inflammatory cells in the PDRN-3 + HMGB-1 group were 18.07 ± 2.10 and 11.75 ± 1.27 on Days 7 and 14, respectively (* *p* < 0.05, *** *p* < 0.001; Figure 5E).

**Figure 6:**
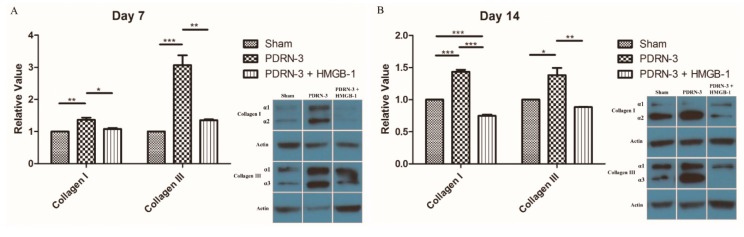
(**A**) Western blots for type I and type III collagen on Day 7. The expression of type I and III collagen was significantly higher in the PDRN-3 group compared with the sham and PDRN-3 + HMGB-1 group (* *p* < 0.05, ** *p* < 0.01, *** *p* < 0.001). (**B**) Western blots for type I and type III collagen on Day 14. The PDRN-3 group also demonstrated higher collagen synthesis compared with the other groups. PDRN accelerated wound healing by promoting early collagen synthesis. This effect of PDRN was reversed by HMGB-1 administration (* *p* < 0.05, ** *p* < 0.01, *** *p* < 0.001).

The synthesis of type I and type II collagen was analyzed by Western blots in the sham, PDRN-3, and PDRN-3 + HMGB-1 groups. On Day 7 of the postoperative period, type I collagen in the PDRN-3 and PDRN-3 + HMGB-1 groups increased by 1.36 ± 0.07-fold and 1.08 ± 0.03-fold, respectively, compared with the sham group (* *p* < 0.05, ** *p* < 0.01; Figure 6A). Type III collagen in the PDRN-3 and PDRN-3 + HMGB-1 groups also increased by 3.07 ± 0.31-fold and 1.35 ± 0.03-fold, respectively, compared with the sham group (** *p* < 0.01, *** *p* < 0.001; Figure 6A). On Day 14 of the postoperative period, type I collagen in the PDRN-3 and PDRN-3 + HMGB-1 groups increased by 1.43 ± 0.03-fold and 0.75 ± 0.02-fold, respectively, compared with the sham group (*** *p* < 0.001; Figure 6B). Type III collagen in the PDRN-3 and PDRN-3 + HMGB-1 groups also increased by 1.38 ± 0.11-fold and 0.88 ± 0.01-fold, respectively, compared with the sham group (* *p* < 0.05, ** *p* < 0.01; Figure 6B). These results indicated that PDRN administration stimulated wound healing by reducing inflammation and increasing collagen synthesis. Furthermore, the effect of PDRN was reversed by HMGB-1 administration.

## 3. Discussion

As an A2AR agonist, PDRN exerts angiogenic effects via vascular endothelial growth factor (VEGF) augmentation [19,20] and tissue-repair effects via fibroblast stimulation [21,22]. Additionally, the activation of A2AR has an anti-inflammatory effect due to the inhibition of several pro-inflammatory mediators [16,18,23]. Previous investigations found that the injection of PDRN until the proliferative phase of wound healing resulted in a fibroplasia effect [20,22,24]. Although a fibroplasia effect from the prolonged injection of PDRN is beneficial to the compromised wound, it is not beneficial with respect to scar formation. Scar formation as the final result of wound healing is due to temporary overlaps of three phases: inflammatory, proliferative, and remodeling. In normal wound healing, there is an influx of inflammatory cells to the wound site until Days 4–6, followed by a proliferative phase during which inflammatory cells are replaced with fibroblasts. The transition between inflammation and proliferation is important because abnormal inflammatory prolongation results in excessive scarring [25]. Thus, we suspected that PDRN, which has both anti-inflammatory and collagen synthesis effects, could be beneficial in scar formation when administered during the inflammatory phase. Because uncontrolled and prolonged inflammation of the dermis produces pathologic scars, reduced inflammation and faster wound healing could have a beneficial effect on scar formation [4]. In this regard, we hypothesized that intensive administration of PDRN during the inflammatory phase for approximately three to seven days post-wounding could reduce inflammation and promote progression to the proliferative phase and early collagen synthesis.

Minimizing inflammation is thought to be associated with reducing scar formation [26,27]. Continuous and histologically localized inflammation of the reticular layer of the dermis produces pathologic scars [4]. Similarly, it has been shown that in surgical wounds, dermal inflammation that persists for 1–2 weeks can result in aberrant scarring and eventually pathologic scars [4]. Thus, we attempted to determine the degree of inflammation after PDRN administration. CD45, which is also referred to as common leukocyte antigen, is a ubiquitous membrane glycoprotein expressed in all hematopoietic cells, except mature erythrocytes [28]. The degree of cellular infiltration was significantly greater in the sham group, and the majority of these cells were CD45+ leukocytes. To objectively analyze the infiltration of inflammatory cells, we counted cell numbers in H&E-stained tissues. The scar areas in the sham group were more abundantly infiltrated with inflammatory cells than those in the PDRN-treated groups. Hence, our results indicated that the administration of PDRN could decrease inflammation, which may be a factor in excessive scar formation.

HMGB-1 is a ubiquitous nuclear protein that exists in eukaryotic cells [29]. Extracellular HMGB-1 regulates the synthesis of monocyte-derived pro-inflammatory cytokines such as TNF-α and IL-1 [30]. Extracellular HMGB-1 secreted from necrotic and inflammatory cells triggers inflammation by inducing inflammatory cell chemotaxis, which in turn initiates the production of pro-inflammatory cytokines by other inflammatory cells [12,31]. In previous investigations, administration of PDRN down-regulated the expression of the inflammatory cytokine HMGB-1 in arthritis and periodontitis models [16,18]. Although the relationship between HMGB-1 and scar formation remains unclear, HMGB-1 induced scar formation when applied to early embryonic murine skin wounds [32]. Thus, we hypothesized that PDRN may down-regulate inflammation and scar formation in surgical wounds by suppressing HMGB-1. On Day 7, extracellular HMGB-1 expression was widespread throughout the granulation tissue in all treatment groups, particularly in the sham group. On Day 14, extracellular HMGB-1 expression remained apparent in the sham group but was decreased in the PDRN treatment groups. Thus, our results implied that PDRN administration reduced HMGB-1 as a potent inflammatory mediator. To confirm the role of HMGB-1 in PDRN action, we administered HMGB-1 to the PDRN-3 group. This additional HMGB-1 administration counteracted the effects of PDRN, resulting in a wide scar area. Furthermore, collagen synthesis was also significantly suppressed by the administration of HMGB-1 to PDRN-treated rats. Inflammatory cell infiltration was also increased on Day 14 in PDRN-treated rats that were administered HMGB-1. Therefore, HMGB-1 could reverse the collagen synthesis and anti-inflammatory effects of PDRN. These results supported our hypothesis that PDRN exerts its anti-inflammatory and collagen synthesis effects via HMGB-1 suppression.

Histologic analyses of tissue samples collected on Day 7 showed that all groups were in the early proliferative phase, as indicated by the formation of granulation tissue; however, substantial inflammatory cell infiltration was observed in the sham group, which continued to exhibit granulation tissue and inflammatory cell infiltration on Day 14. Furthermore, the scar widths were significantly narrower in the PDRN-treated groups than in the sham group. PDRN could reduce the granulation tissue that serves as potential scar tissue during the early wound-healing phase. Thus, PDRN prevents scar formation via the promotion of fast wound healing by suppressing inflammation and enhancing collagen synthesis.

In summary, we concluded that faster wound healing and decreased scar formation were induced by the anti-inflammatory and collagen synthesis effects of PDRN. However, the short experimental period could be a potential limitation of our study. Nonetheless, it was obvious that the reduced formation of granulation tissue and decreased infiltration of inflammatory cells combined with faster wound healing following PDRN administration could improve the characteristics and sizes of scars.

## 4. Materials and Methods

### 4.1. Animal Model

Twenty-four male Sprague-Dawley (SD) rats were used to study incisional wounds. All animal protocols used in this study were approved by the Yonsei University Institutional Animal Care and Use Committee (16 April 2014). General anesthesia was induced via intraperitoneal injection of a zolazepam tiletamine mixture (30 mg/kg, Zoletil^®^; Virbac, Carros, France) and xylazine (10 mg/kg, Rompun^®^; Bayer, Leverkusen, Germany). A 6 × 1 cm^2^ rectangular design was made to excise the skin and the panniculus carnosus muscle. The skin and panniculus carnosus muscle were excised, and only the skin layer was closed to maximize tension stress by leaving the muscle layer unrepaired. After surgery, the rats were randomly assigned to one of three treatment groups: sham (*n* = 8), PDRN-3 (*n* = 8), and PDRN-7 (*n* = 8). The sham group was injected with 1 mL of normal saline for seven days, whereas the PDRN-3 and PDRN-7 groups were administered PDRN via intraperitoneal injection (8 mg/kg, Placentex Integro^®^, Mastelli SRL, Sanremo, Italy) for three and seven days, respectively.

Another experiment was performed to clarify whether the effects of PDRN on scar diminishing and inflammation were mediated by HMGB-1. To determine whether HMGB-1 administration increased scar formation and inflammatory cell infiltration, 400 μg of HMGB-1 (HMGBiotech, Milan, Italy) diluted in 500 μL of normal saline was administered on a central 2-cm area of the incisional wound via intradermal injection for three days followed by PDRN administration as described for the PDRN-3 group (PDRN-3 + HMGB-1 group; *n* = 6). HMGB-1 was administered before intraperitoneal injection of PDRN. Other experimental protocols were the same as described above.

### 4.2. Histologic Analysis

Four SD rats in each group were euthanized on Days 7 and 14, and tissue biopsies were performed to evaluate inflammatory cell counts and scar areas. Tissue samples (10 mm thick) were obtained from the middle region of the wound, where there was maximal tension. All tissues were fixed in 10% neutral buffered formalin, embedded in a paraffin block, and stained with H&E and M-T stain.

The H&E- and M-T-stained tissues were examined under a light microscope at 40× to estimate the scar areas and the degree of tissue granulation. The scar area was estimated using ImageJ^®^ software version 1.49 (National Institutes of Health, Bethesda, MD, USA). In each wound, the scar and/or granulation tissue areas were obtained from two tissue sections representing different areas of the same wound. The mean scar and/or granulation tissue areas for each wound were then converted from pixel numbers to square micrometers that were calculated using the ratio of pixel numbers to the scale bar.

The H&E-stained tissues were also examined using a light microscope at 400× to evaluate the degree of inflammatory cell infiltration in the scar tissue. The inflammatory cells were counted in four serial sections of tissue from within the dermis of the scar area. The numbers of inflammatory cells were calculated for each wound from two tissue sections representing different areas of the same wound, and then the mean number of inflammatory cells was obtained.

### 4.3. Immunohistochemistry for HMGB-1

Tissues obtained from the middle region of the wound were fixed with 10% formaldehyde and embedded in a paraffin block. Tissue sections were pretreated with a 3% hydrogen peroxide solution for 10 min to block endogenous peroxidase activity and then treated with a protein blocking serum-free reagent (X0909 DAKO, Carpinteria, CA, USA) for 30 min to prevent non-specific reactions. The sections were incubated at 4 °C overnight with primary antibodies (HMGB-1, Abcam, Cambridge, MA, USA) and then incubated at room temperature for 20 min with secondary antibodies from the DAKO Envision Kit (DAKO, Carpinteria, CA, USA). The expression of HMGB-1 in the scar area was semi-quantitatively analyzed using the MetaMorph^®^ image analysis software version 7.8 (Universal Imaging, West Chester, PA, USA).

### 4.4. Immunofluorescence Assay for CD45

For immunofluorescence microscopy, the samples were blocked with 1% bovine serum albumin (BSA) followed by incubation with anti-CD45 (Life Technologies Co., Carlsbad, CA, USA) overnight at 4 °C. The next day, the cells were washed in phosphate-buffered saline and incubated with an Alexa Flour 488-conjugated goat anti-rabbit immunoglobulin G secondary antibody for 60 min at room temperature. The final antibody treatment also contained tetramethylrhodamine isothiocyanate–conjugated phalloidin and 4′,6-diamidino-2-phenylindole stain (DAPI; both at 1 g/mL; Sigma, St. Louis, MO, USA) for nuclear staining. The slides were mounted in Vectashield^®^ HardSet Mounting Medium with DAPI (Vector Laboratories, Burlingame, CA, USA), and the cells were viewed under a confocal laser scanning microscope (LSM700; Carl Zeiss MicroImaging, Thornwood, NY, USA)

### 4.5. Western Blots for Type I and Type III Collagen

The wound homogenates were analyzed via Western blotting. The protein concentration was quantified using a bicinchoninic acid assay (Thermo Fisher Scientific, Waltham, MA) and normalized to a standard concentration using extraction buffer. The proteins were separated by sodium dodecyl sulfate-polyacrylamide gel electrophoresis (SDS-PAGE) and then transferred to polyvinylidene difluoride membranes (Millipore, Billerica, MA, USA). The membranes were blocked for 1 h with 3% BSA in 1X TBST and then incubated overnight at 4 °C with monoclonal mouse anti-collagen type I α1 and α2 antibodies and anti-collagen type III α1 and α3 antibodies (1:1000, Abcam, Cambridge, MA, USA). The primary antibody was detected using a horseradish peroxidase-conjugated goat anti-mouse or anti-rabbit secondary antibody (1:5000, Cell Signaling, Beverly, MA, USA). The protein bands were visualized using an ECL detection kit (Thermo Fisher Scientific, Waltham, MA, USA) according to the manufacturer’s instructions. Finally, immunoblot signals were analyzed using ImageJ^®^ software version 1.49 (National Institutes of Health, Bethesda, MD, USA). The results from each group were expressed as the integrated intensity relative to the sham group, measured with the same batch.

### 4.6. Statistical Analysis

Each measurement is shown as the mean ± SEM. All pairwise differences between the group measurements were examined by independent and paired t-tests using standard software (SPSS for Windows v15.0; SPSS Inc., Chicago, IL, USA). Statistical significance was set at *p* < 0.05.
